# Pathogenesis of Chronic Plaque Psoriasis and Its Intersection With Cardio-Metabolic Comorbidities

**DOI:** 10.3389/fphar.2020.00117

**Published:** 2020-02-25

**Authors:** Paolo Gisondi, Francesco Bellinato, Giampiero Girolomoni, Cristina Albanesi

**Affiliations:** ^1^Section of Dermatology and Venereology, Department of Medicine, University of Verona, Verona, Italy; ^2^Laboratory of Experimental Immunology, Istituto Dermopatico dell'Immacolata, IDI-IRCCS, Rome, Italy

**Keywords:** psoriasis, pathogenesis, treatment, cardio-metabolic comorbidities, inflammation

## Abstract

Psoriasis is a chronic, systemic immune-mediated disease characterized by development of erythematous, indurated, scaly, pruritic plaques on the skin. Psoriasis is frequently associated to comorbidities, including psoriatic arthritis, cardiovascular diseases, diabetes mellitus, obesity, non-alcoholic fatty liver disease, and inflammatory bowel diseases. In this review, we discuss the pathophysiological relationship between psoriasis and cardio-metabolic comorbidities and the importance of therapeutic strategies to reduce systemic inflammation in patients with moderate-to-severe psoriasis. Pathogenesis of psoriasis and its comorbidities share both genetic predisposition and inflammatory pathways, which include the TNFα and the IL-23/IL-17 pathways. These pathways are selectively addressed by biological treatments, which have substantially changed the outcomes of psoriasis therapy and affect positively comorbidities including reducing cardiovascular risk, allowing a more comprehensive approach to the patient.

## Introduction

Psoriasis is a chronic, inflammatory disease involving skin of genetically predisposed individuals. It affects approximately 2% of the general population, with >50% of patients presenting in the first three decades of life (Bowcock, 2005; Griffiths and Barker, 2007; Chandran and Raychaudhuri, 2010). There is a wide spectrum of cutaneous manifestations of psoriasis, with individual lesions varying from pinpoint to large plaques, or even generalized erythroderma (Griffiths and Barker, 2007). The most common and well-recognized morphological presentation of psoriasis is the plaque type. These lesions reflect skin inflammation, epidermal hyperplasia, and angiogenesis, as consequence of a dysregulation of skin immune responses (Griffiths and Barker, 2007; Lowes et al., 2014). However, altered immunity can operate systemically and signs of inflammation can readily be detected at areas outside the skin. As a result, in psoriatic patients, inflammation is widespread, and, in most cases, different comorbid conditions co-exist (Binus et al., 2012; Armstrong et al., 2013a; Takeshita et al., 2017; Boehncke, 2018). Among them, cardio-vascular diseases (CVD) are importantly associated to psoriasis, together with diabetes mellitus and metabolic disorders, including obesity, hypertension, dyslipidemia, and non-alcoholic fatty liver disease (NAFLD) (Armstrong et al., 2012; Langan et al., 2012; Armstrong et al., 2013a; Armstrong et al., 2013b; Coto-Segura et al., 2013; Armstrong et al., 2015; Candia et al., 2015; Takeshita et al., 2017). Also inflammatory bowel diseases (IBD) and kidney diseases, as well as infections, depression, and cancer are often comorbid conditions that can be developed in psoriatic patients (Bernstein et al., 2005; Binus et al., 2012; Wan et al., 2013). To date, it is still controversial whether the chronic inflammatory nature of psoriasis is a contributing factor or an independent risk factor for the development of these comorbidities. Consistently, inflammatory arthritis, that frequently affects small joints of psoriatic patients, could be considered either as an extracutaneous manifestation of psoriasis or as a separate entity, and thus a comorbidity (Gladman et al., 2005). Among the comorbid conditions, cardiovascular and metabolic diseases are of particular importance, as they may considerably reduce life expectancy of psoriatic patients, especially of those affected by the most severe forms of the disease (Armstrong et al., 2013a; Samarasekera et al., 2013).

This review summarizes the evidence on the pathophysiological relationship between psoriasis and its comorbidities, with emphasis on cardio-metabolic comorbid conditions. The ability of biologic therapies to reduce systemic inflammation and to ameliorate comorbidities, including reducing cardiovascular risk, in patients with psoriasis will be also discussed.

## Pathogenesis of Chronic Plaque Psoriasis

Primary cause of psoriasis is a dysregulation of immune responses, which manifests in individuals carrying one or more psoriasis susceptibility genes, either skin specific or related to immune functions, and after their exposure to certain environmental triggers (Nestle et al., 2009; Perera et al., 2012; Di Meglio et al., 2014). The latter include physical trauma (Koebner phenomenon) and infections, which trigger innate immune responses by promoting the formation and the release of nucleic acid/autoantigen complexes by injured skin cells. In particular, complexes formed by the cathelecidin LL37 and self-DNA/RNA fragments activate plasmacytoid dendritic cells (pDCs), a subset of DC releasing high IFN-α and TNF-α (Nestle et al., 2005; Lande et al., 2007) pDC recruitment in psoriatic skin is determined by the chemokine chemerin, and correlates with the massive presence in the mid-papillary dermis of other innate immune cells, such as neutrophils, macrophages, monocytes, and mast cells (Albanesi et al., 2009). Local production of IFN-α and other type I IFNs induces keratinocyte immune activation and maturation of myeloid DCs (mDCs), with consequent beginning of the adaptive immune response phase. As a consequence, a IL-23/IL-17 inflammatory environment is established in psoriatic skin, with DC and macrophage-derived IL-23 promoting the type 17 helper (Th17) and cytotoxic (Tc17) cell effector functions (Lowes et al., 2013; Girolomoni et al., 2017). In parallel, mDCs induce the IL-12/IFN-γ cytokine axis, which is responsible for the strong type II IFN transcriptomic signature and the high frequency of Th1 and Tc1 cells in both psoriasis plaques and peripheral blood of patients (Schlaak et al., 1994; Austin et al., 1999). The innate lymphoid cells (ILC), a class of immune cells bearing lymphoid morphology, but no immune cell lineage markers (Spits and Cupedo, 2012; Bernink et al., 2013), together with γδ-T cells, an innate-like T-cell population involved in surveillance of epithelial surfaces, are also critical contributors to plaque development by releasing considerable levels of IL-17 and IL-22 (Villanova et al., 2014). Also mast cells and neutrophils can represent an innate sources of IL-17 in psoriatic skin (Lin et al., 2011).

Following the massive expansion of effector immune cells in both the epidermis and dermis, very high levels of IL-17 and IL-22 are produced. These two cytokines, together, mediate most of the epidermal hyperplasia by impairing differentiation of keratinocytes, and inducing their premature maturation and aberrant cornification (Nograles et al., 2008). IL-17 also functions by activating keratinocytes to produce neutrophil- and T-cell-recruiting chemokines, namely CXCL1/CXCL2/CXCL8 and CCL20, respectively, as well as antimicrobial peptides (AMP), including LL37 and S100 family members (Albanesi et al., 1999; Wilson et al., 2007). Thus, IL-17 is central in a pathogenic loop linking T cells and keratinocytes. On the other hand, the T-cell-derived IFN-γ and TNF-α activate a plethora of inflammatory pathways in resident skin cells, in particular keratinocytes and endothelial cells (Albanesi and Pastore, 2010; Albanesi et al., 2018). Each cytokine regulates distinct responses with a certain degree of synergism in getranscription factor regulating gene ene expression induction/inhibition. Most of the effects induced by IFN-γ are potentiated by TNF-α, which intracellularly activates NF-κB, a transcription factor regulating gene expression frequently in collaboration with the signal transducer and activator of transcription (STAT)1 induced by IFN-γ. TNF-α induces expression of ICAM-1 on resident skin cells, permitting the adhesion and extravasation of circulating leukocytes. Moreover, TNF-α stimulates the production of several chemokines active on immune cells, as well as pro-inflammatory cytokines, in particular IL-6 and IL-1, which sustain Th17 expansion (Albanesi et al., 2018; Chiricozzi et al., 2018). Importantly, TNF-α, together with IL-17, induces IL-36γ in psoriasis lesions, which in turn promotes expression of AMP and chemokines recruiting neutrophils and Th17 cells, as well as interferes with terminal differentiation and cornification process of the epidermis (Carrier et al., 2011). Interestingly, transcriptional profiling studies conducted on lesional psoriatic skin showed that the IFN-γ-signature predominates, even though IL-17 and TNF-α also potently induce a vast panel of genes (Nograles et al., 2008; Chiricozzi et al., 2014). Although studies demonstrated a central role of IL-22 in psoriasis pathogenesis by activating STAT3-dependent genes involved in differentiation and proliferation processes, this cytokine induces a limited panel of genes compared to IL-17, as detected in human lesional psoriatic skin (Chiricozzi et al., 2014). Importantly, intrinsic or genetic alterations of keratinocytes in the activation of key signaling pathways induced by pro-inflammatory cytokines (i.e., STAT1 and STAT3, NF-κB, ERK1/2, and Act1) may be responsible for the typical unbalance between proliferation and differentiation processes, as well as inflammatory signatures observed in psoriatic epidermis (Harden et al., 2015; Capon, 2017).

Collectively, a pathogenic cross-talk between DCs, T cells, and keratinocytes, sustained by type I IFNs, IL-23, IL-12, IFN-γ, IL-17, TNF-α, and IL-22, and possibly supported by other immune cell players, causes keratinocyte production of pro-inflammatory molecules, as well as concurs to derange proliferative and differentiative programs of the epidermis. This becomes a self-amplifying loop, where these products and altered homeostasis act back on T cells and DC to perpetuate the cutaneous inflammatory processes.

## Comorbidities of Chronic Plaque Psoriasis

Since 1897, when Strauss reported an association between psoriasis and diabetes, emerging epidemiologic studies find additional associations between psoriasis and inflammatory diseases, apart from well-known psoriatic arthritis (PsA) (Strauss, 2009). The association between psoriasis and inflammatory diseases is stronger in the most severe forms of psoriasis (Takeshita et al., 2017). Comorbidity psoriasis burden includes mainly CVD, metabolic disorders, such as diabetes, dyslipidemia, and metabolic syndrome, inflammatory bowel disease, and kidney disease. The prevalence of traditional CV risk factors such as hypertension, obesity, diabetes, dyslipidemia, metabolic syndrome, and cigarette smoking is increased in patients with psoriasis compared to controls (Gisondi et al., 2010).

**Figure 1:**
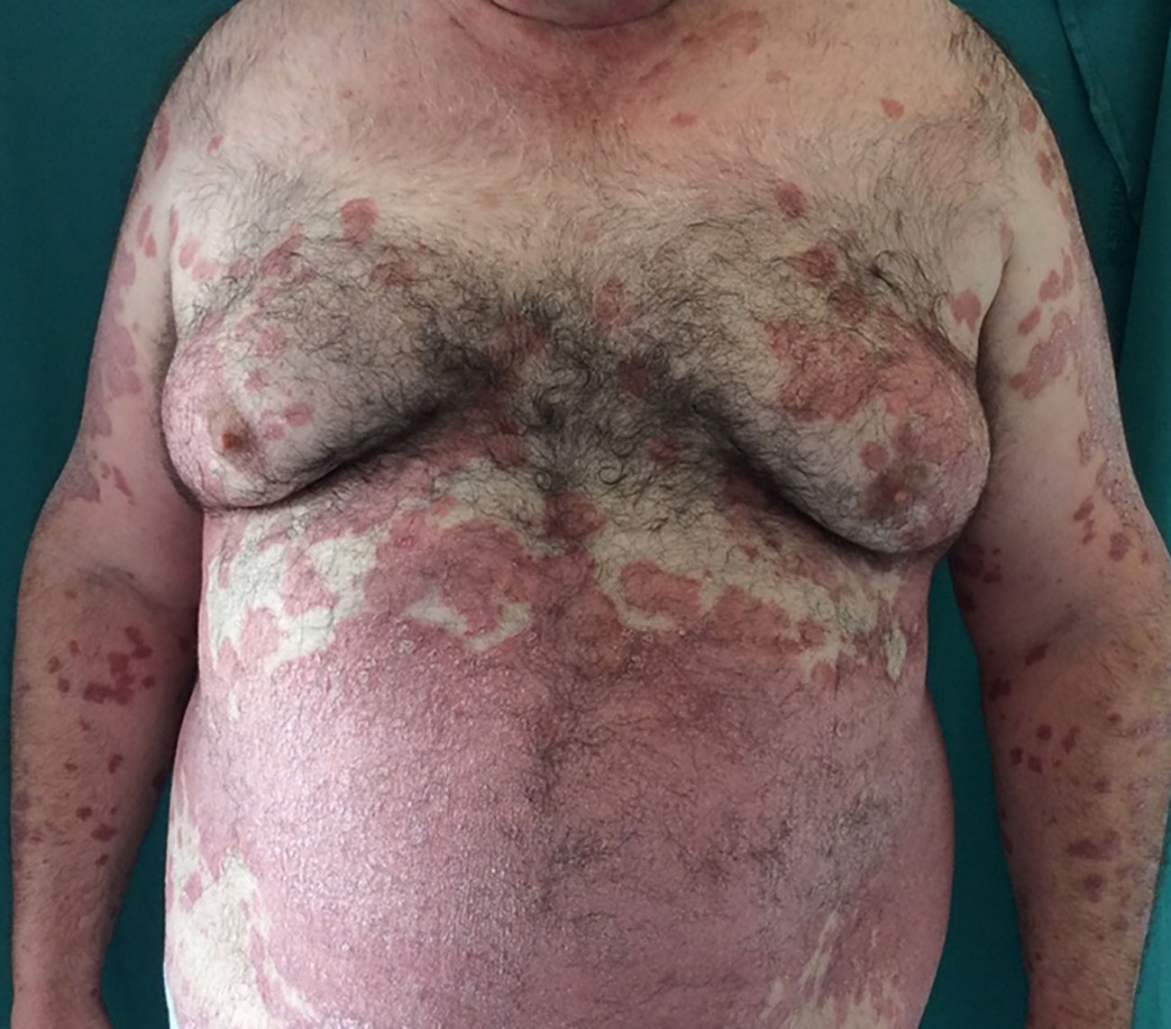
Man affected by psoriasis and central obesity.

Patients with psoriasis are more frequently overweight or obese (**Figure 1**). Obesity, but also BMI, hip circumference and waist-hip ratio are independent risk factors for psoriasis. The risk was found to increase with obese severity, as higher body mass index (BMI) (Kumar et al., 2013). A meta-analysis of 16 observational studies found a pooled OR of 1.66 for the association between the two diseases (95% CI 1.46–1.89) (Armstrong et al., 2012). A cross-sectional study found a direct correlation between psoriasis severity and obesity (Duarte et al., 2013). As obesity, also hypertension is prevalent among psoriatic patients compared to those who are not affected. A meta-analysis of 24 studies showed a pooled OR of 1.58 (95% CI 1.42–1.76) for the association between hypertension and psoriasis (Armstrong et al., 2013b). Poor controlled and sever hypertension appear to increase with more severe disease (Langan et al., 2012). Psoriasis is an independent risk factor for diabetes, with higher risk with greater severity of psoriasis (Takeshita et al., 2015). Diabetic patients with psoriasis appear to be more likely to suffer from micro and macro-vascular complications, compared to patients without psoriasis. The pooled OR for psoriasis associated with diabetes in a meta-analysis of 44 studies was 1.76 (95% CI 1.59–1.96) (Coto-Segura et al., 2013). Atherogenic lipid profile and reduced high density lipoprotein (HDL) were reported among patients with psoriasis, compared to patients without psoriasis. Dyslipidemia may be more prevalent in psoriatic patients. In a systematic review, most of the studies found significant association between psoriasis and dyslipidemia, with OR ranging between 1.04 and 5.55 (Ma et al., 2013). Higher odds of dyslipidemia were reported in severe psoriasis, compared to patients with mild disease. According to some studies, dyslipidemia may be a risk factor for developing psoriasis (Wu et al., 2014). Metabolic syndrome comprises a group of well-known cardiovascular (CV) risk factors, including central obesity, hypertension, insulin resistance, and dyslipidemia. A cross-sectional study reported that the prevalence of metabolic syndrome correlated directly with psoriasis body surface area (Langan et al., 2012). A meta-analysis of 12 studies found a pooled OR of 2.26 (95% CI 1.70–3.01) for the association with psoriasis (Armstrong et al., 2013c). The analysis of the separate components of metabolic syndrome showed the strongest association between obesity, suggesting that the adiposity is the main factor in the association between psoriasis and metabolic syndrome.

Although both CV risk factors and CVD are prevalent among psoriatic patients, psoriasis is an independent risk factor for the latters. A large cohort study found that psoriasis is an independent risk for myocardial infarction (MI), also considering other traditional CV risk factors (Gelfand et al., 2006). Two meta-analyses showed that the risks of MI, stroke, and death caused by CVD, collectively termed as major cardiovascular events, is greatest among patients with psoriasis and appear to be greatest among those with severe or longer duration disease (Armstrong et al., 2013a; Samarasekera et al., 2013). Psoriasis, as an independent CV risk factor, was reported to strongly impact on the Framingham Risk Score for more than 60% of the patients (Mehta et al., 2012).

The epidemiology of the relationship between IBD and psoriasis is still unclear. Many studies reported that psoriasis may be associated with an increased incidence and prevalence of IBD (and *vice versa*), in particular Crohn's disease (Bernstein et al., 2005; Cohen et al., 2009). Psoriasis may be more strongly associated with Crohn's disease than ulcerative colitis withOn the other hand, the T-cell-derived ORs of 2.49 (95% CI 1.71–3.62) *versus* 1.64 (95% CI 1.15–2.23), respectively (Mehta et al., 2012). Patients with psoriasis and concomitant IBD have a higher rate of comorbidities (seronegative arthritis, thyroiditis, diabetes, and lymphoma) than patients with only psoriasis (Binus et al., 2012). Considering the potentially hepatotoxicity and nephrotoxicity of many psoriatic treatments, there is a great interest in the epidemiology of liver and renal disease in psoriatic patients. NAFLD is a common liver disease comprising mild forms of steatosis up to steato-hepatitis. Psoriasis is frequently associated to metabolic disorders that can favor liver steatosis. The prevalence of NAFLD among patients with psoriasis is greater compared with non-psoriatic patients, but the evidence of the association between psoriasis and hepatic diseases is based on seven low-to-moderate quality observational studies with pooled OR of 2.15 (95% CI 1.57–2.94) (Candia et al., 2015). Moderate-to-severe psoriasis may be an independent risk factor for chronic kidney disease (CKD) and end-stage renal disease. A cohort study found that severe psoriasis may be associated with CKD and end-stage renal disease with HRs of 1.93 (95% CI 1.79–2.08) and 4.15 (95% CI 1.70–10.11), respectively (Wan et al., 2013).

Several studies have reported association between psoriasis and other emerging comorbidities such as cancer, especially T-cell lymphoma, mood disorders, pneumopathies such as chronic pulmonary disease and obstructive sleep apnea, peptic ulcer disease, hyperuricaemia/gout, osteoporosis, and sexual dysfunction (Takeshita et al., 2017). Some of these need to be confirmed in larger studies.

## Pathogenesis Behind the Comorbidities in Psoriasis

**Figure 2:**
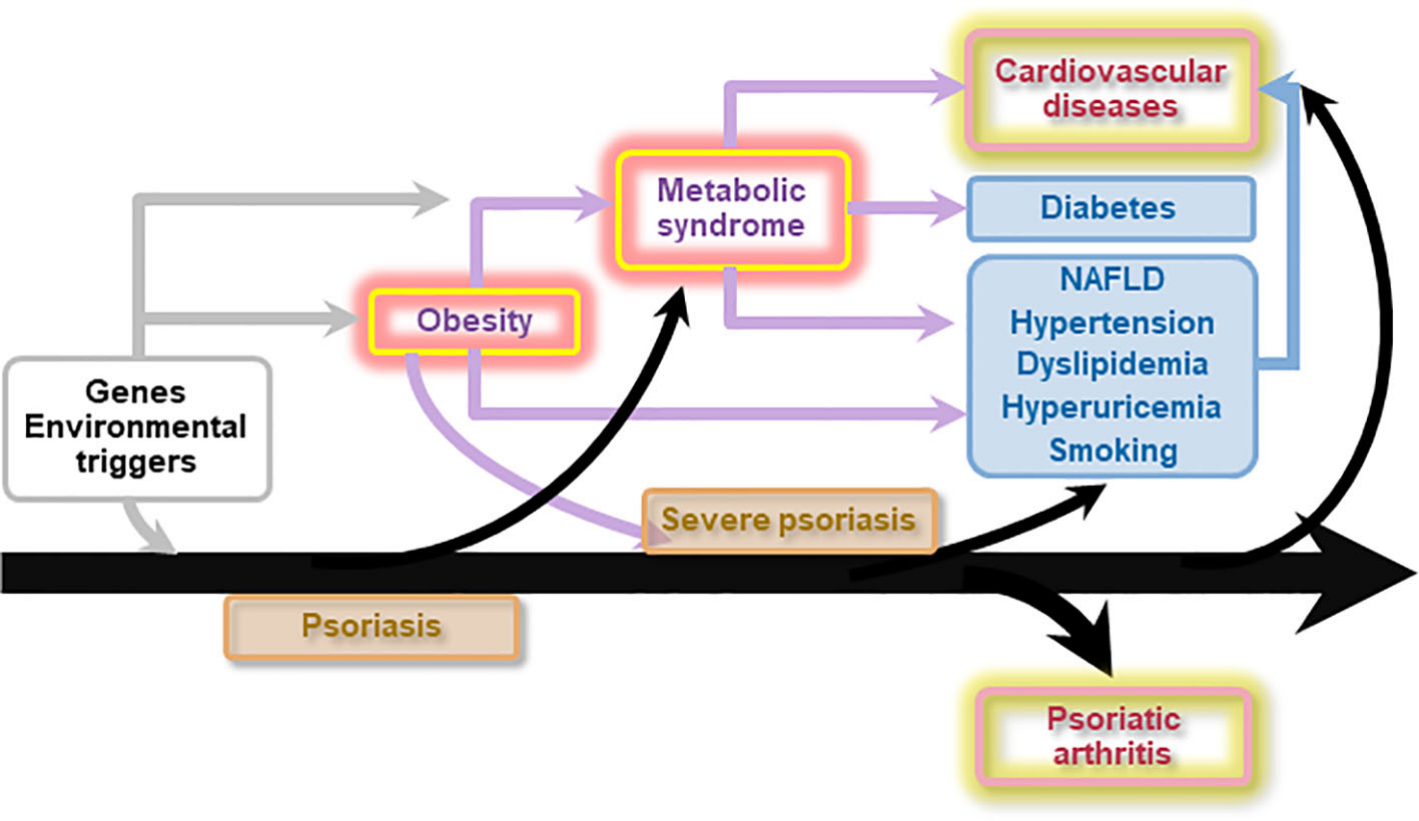
Genetic and environmental factors predispose to psoriasis and obesity. Obesity is a risk factor for both psoriasis and metabolic syndrome. However, inflammation associated with moderate to severe psoriasis can in turn favor insulin resistance, dyslipidemia, obesity, and non-alcoholic fatty liver disease (NAFLD), hence directly and/or indirectly fuelling atherosclerosis, and configuring the so-called “psoriatic march”. Ultimately, moderate to severe psoriasis directly and indirectly increases the risk of cardiovascular diseases and mortality. Psoriasis also precedes the development of psoriatic arthritis.

The pathogenesis behind psoriasis comorbidity remains partially unknown; however different factors may be involved, including common pattern of immune responses and inflammatory pathways, shared risk factors, and genetic predisposition (Takeshita et al., 2017) (**Figure 2**).

Patients with psoriasis are enriched for certain common genetic variants (HLA, FUT2, UBE2L3, SH2B3) that predispose to increased risk of dyslipidemia, hypertension, and CVD (Lu et al., 2013).

Most common inflammatory pathways between psoriasis and its comorbidities strictly depends on the expansion of circulating pathogenic T cells, instructed by DC activated at skin sites, and to the establishment of systemic inflammation. These pathways involve key cytokines and signal transducers, such as IL-23R, IL-12B, IL-21, IL-4, and IL-5, in psoriatic arthritis; IL-23R, IL-12B, IL-13, Rel, TYK2, and JAK2 in Crohn's disease (Ellinghaus et al., 2012; FitzGerald et al., 2015; Veale and Fearon, 2018). In addition to common cytokine hallmarks, psoriasis and cardiometabolic diseases may share other mutations, such as CDKAL1 and apolipoprotein E (Eiris et al., 2014).

A high number of studies have shown that psoriasis and cardiometabolic disorders have rather more commonly similar underlying immunologic mechanisms related to Th1 and Th17 cells activation (Lockshin et al., 2018). Inflammatory mediators released from psoriatic lesions, including TNF-α, IFN-α, IFN-γ, IL-1, IL-6, and IL-17, may have systemic effects contributing to atherogenesis. Consistently, recent studies conducted on human tissues showed that psoriasis and atherosclerosis exhibit significant overlap of their transcriptomes and in particular those dependent on TNF-α and IFN-γ, thus providing the linking between the two diseases (Mehta et al., 2017). By contrast, IL-17A and CCL20 genes were higher in psoriasis than in atherosclerosis tissue, whereas IL-17R was expressed at comparable levels. Because of the link between IL-17 and neutrophil infiltration in atherosclerotic plaques and its pathogenic role in psoriasis, it has been suggested that the IL-17/neutrophil axis could take part to atherogenesis associated with psoriatic disease (Sanda et al., 2017). Consistently, aortic vascular inflammation in psoriatic patients has been found to correlate with disease severity and high levels of S100A8/A9 neutrophil activation markers (Naik et al., 2015). In addition, the neutrophil extracellular traps (NET)osis, a defense mechanism operating in psoriasis and based on the formation of cytosolic granule proteins containing autoantigens, has been found to induce macrophage priming, Th17 activation, and immune cell recruitment in atherosclerosis similarly to psoriasis (Aldabbous et al., 2016; Delgado-Rizo et al., 2017; Doring et al., 2017). NET are also shown to directly induce endothelial dysfunction and plaque rupture in human carotid plaque sections (Quillard et al., 2015). Monocyte and neutrophil damage, increased oxidative stress, endothelial dysfunction, angiogenesis, and increased circulating micro particles are other common shared alterations (Quillard et al., 2015). Psoriasis and atherosclerosis patients also share dysfunctional peripheral T regulatory (Treg) cells, a subset of T lymphocytes highly releasing TGFβ, IL-10, and IL-35, with inhibitory function on T cell activation and proliferation, and anti-inflammatory roles through endothelial cell modulation (Sugiyama et al., 2005; Kagen et al., 2006; Takeshita et al., 2014; Meng et al., 2016). In psoriasis, this impairment may be dependent on high IL-6 levels, as demonstrated by blocking IL-6 in co-cultures of Treg cells and effector T cells from psoriatic patients (Goodman et al., 2009).

Systemic inflammation associated with psoriasis also promotes inflammation in the adipose tissue, which harbors cells and molecular components of the immunity system. Psoriatic adipose tissue contains immune cells that can influence cardiometabolic disease (Rose et al., 2014). Among them, T cells, DCs, neutrophils, mast cells, and adipose tissue macrophages contribute to obesity and insulin resistance, whereas eosinophils and Treg are protective. Obesity is also associated to systemic inflammation because of the release of adipokines, including chemerin, adiponectin, resistin, visfatin, C-reactive protein released by macrophages, and T cells infiltrating visceral adipose tissue. Adipokines can contribute to the pathogenesis of insulin resistance and fuel inflammation associated with psoriasis (Davidovici et al., 2010). Adipokines together with chemokines (i.e., CXCL8 and CCL2) produced by visceral adipose tissue can also contribute to progression of atherosclerosis and CDV disease development by influencing endothelial cell function and interaction with immune cells (Henrichot et al., 2005; Karastergiou et al., 2010; Britton and Fox, 2011). An association between obesity and PsA has been confirmed, and the presence of metabolic syndrome and related adipokines correlates with skin and joint disease activity (Eder et al., 2013). However, levels of adipokines have been found to not differ between PsA patients with clinical evident psoriasis and PsA sine psoriasis, reinforcing the concept that metabolic manifestations during psoriatic disease may be independent of severity of cutaneous and articular involvement and are potentially related to the subclinical chronic inflammation (Caso et al., 2019). As abdominal visceral fat, also epicardial adipose tissue has been shown to be increased in patients with psoriasis, and potentially contribute to increased CV risk (Torres et al., 2015). Additionally, epicardial adipose tissue has been referred as potentially responsible for a distinctive pattern of CV disorders seen in psoriasis (accelerated coronary atherosclerosis leading to myocardial infarction; atrial myopathy leading to atrial fibrillation and thromboembolic stroke, and ventricular myopathy leading to heart failure with a preserved ejection fraction) (Packer, 2019). Psoriasis-related inflammation could trigger the progression from normal liver to NAFLD. Pro-inflammatory cytokines and adipokines, including TNF-α, play a pivotal role in pathogenesis of both psoriasis and NAFLD as well as in progression of NAFLD to NASH. Moreover, obesity induces a bio-mechanical stress that may be a possible trigger for PsA (Mantovani et al., 2016).

Finally, as psychosocial impact of psoriasis is relevant, this could favor unhealthy lifestyles, such as alcoholism and smoking habit that are well-known CV and metabolic risk factors. Anxiety, depression, and suicidal ideation and behavior (SIB) are prevalent in patient with psoriasis. There is growing evidence that inflammation is associated with pathophysiology of depression. Pro-inflammatory cytokines such as IL-1 and IL-6 are elevated both in psoriasis and depression. Cytokine-mediated systemic inflammation may be an underlying patho-mechanism of both psoriasis and mental health disorders, such as depression and SIB (Wu et al., 2018a).

## Systemic Treatment of Psoriasis Could Ameliorate Cardiovascular Comorbidities

New psoriasis treatment paradigms have gone beyond the belief of psoriasis as a disease limited to the skin. Borrowing the experience from the studies of other immune-mediated inflammatory diseases, such as Crohn's disease and rheumatoid arthritis, the goals of treating systemic inflammation in psoriasis are two: to prevent and even to reverse comorbidities (Takeshita et al., 2017; Korman, 2019).

Many surrogate laboratory and radiologic biomarkers of inflammation and endothelial dysfunction have been identified among patients with inflammatory conditions. These include C-reactive protein (CRP), erythrocyte sedimentation rate (ESR), levels of glycoprotein acetylation, coronary flow reserve (CFR), flow-mediated dilatation, carotid intima media thickness, vascular inflammation measured through 18-fluorodeoxyglucose positron emission tomography-computed tomography (18F-FDG PET/TC) (Vadakayil et al., 2015; Puig, 2017). For example, in patients with moderate to severe psoriasis treated with systemic therapies studies have reported reduction in ESR and/or CRP levels (Popa et al., 2005). A prospective study found that improvement in Psoriasis Area Severity Index (PASI) score *via* TNF-α inhibitors was associated with reduced aortic vascular inflammation measured using 18F-FDG PET/TC (Bissonnette et al., 2013). A study on 37 patients treated with TNF-α inhibitors for an average of 6 months reported a significant increase in the value of CFR in the left anterior descending coronary (Piaserico et al., 2016). Recently, biologic therapy was shown to be associated with favorable modulation of coronary plaque indices by coronary computed tomography angiography in patients with severe psoriasis (Elnabawi et al., 2019). There are some evidences in favor of the hypothesis that treating psoriasis with systemic agents could prevent CVD, as a result of suppression of systemic inflammation. In a recent meta-analysis in patients with psoriasis and PsA, systemic therapy was found to significantly decrease the risk of all cardiovascular events with a RR of 0.75 (95% CI 0.63–0.91). With the exception of methotrexate, there are no studies formally evaluating the effect of any anti-psoriatic therapy as a treatment for coronary heart disease (Roubille et al., 2015). Two retrospective analysis of cardiovascular events rates in patients with psoriasis found that patients receiving rouTNF-α inhibitors had significant lower risks for MI compared with patients receiving topical therapies with OR 0.5 (95% CI 0.32–0.79) or phototherapy, HR 0.77 (95% CI 0.60–0.99) (Wu et al., 2012; Wu et al., 2018b). Few studies found no significant differences in overall MI risk between patients treated with systemic therapies and those who received ultraviolet B phototherapy. In a randomize double-blind clinical trial adalimumab reduced key markers of inflammation including glycoprotein acetylation compared with phototherapy, with no effect on glucose metabolism and vascular inflammation (Mehta et al., 2018). The protective cardiovascular effect could be not exclusive to TNF-α inhibitors. CARIMA study indicates that secukinumab might have a beneficial effect on cardiovascular risk by improving the endothelial function (von Stebut et al., 2019).

Obesity is accompanied by a dysregulation of adipocytokines and systemic inflammation and has a well-known effect on psoriasis severity and response to therapies. Current data suggest that weight loss improves psoriasis. Meta-analysis of three randomized control trials confirmed that weight loss following lifestyle interventions (diet or physical activity) improves psoriasis compared with reduction in the PASI score with a pooled mean difference of -2.49 (95% CI -3.90 to -1.08; *P* = 0.004) (Mahil et al., 2019). Long-term weight loss in patients with psoriasis has long-lasting positive effects on the severity of psoriasis (Gisondi et al., 2016). A possible role for biologic agents in reducing obesity in psoriasis has not been observed to date. Although TNF-α inhibitors can induce modest weight gain, they do not cause an increase in visceral adipose tissue and thee association between low-carbohydrates calorie-restricted diet and anti-TNF-alpha therapy seems to be able to improve the anthropometric profile of psoriasis patients (Campanati et al., 2017). No evidence of clinically weight gain has been observed in studies of ustekinumab or ixekizumab. Since IL-17A does not alter adipogenesis and/or insulin resistance mediated by an inflammatory environment and contributes only to the propagation of inflammation in human obese adipose tissues, anti-IL17A agents may play a beneficial effect in inflammatory pathologies, where obesity contributes to poorer response to biologic treatments (Pestel et al., 2019).

## Conclusions

Psoriasis is increasingly being recognized as a systemic inflammatory disorder affecting not only skin and joints. The association with metabolic disorders, such as diabetes, dyslipidemia, and metabolic syndrome, and CVD deserves special attention. Common genetics and shared immuno-inflammatory pathways may partially explain this association. Cutaneous lesions produce a wide range of inflammatory products that are released into systemic circulation and fuel the systemic inflammation. Other non-cutaneous sites, like adipose tissue, can contribute to the inflammatory state. Systemic therapies targeting psoriasis may prevent and even reverse cardio-metabolic comorbidities as a result of suppression of systemic inflammation. It is important for clinicians to recognize psoriasis comorbidity burden to ensure a comprehensive medical care for the patients. In the view of psoriasis as systemic inflammation disease new treatment paradigms may potentially reduce or prevent the comorbidities associated with systemic inflammation.

## Author Contributions

Each author has contributed in the ideation and writing of the manuscript, and each author has checked the final version of the paper.

## Conflict of Interest

The authors declare that the research was conducted in the absence of any commercial or financial relationships that could be construed as a potential conflict of interest.
